# Influence of miR-221/222 on cardiomyocyte calcium handling and function

**DOI:** 10.1186/s13578-021-00676-4

**Published:** 2021-08-17

**Authors:** Maria Knyrim, Sindy Rabe, Claudia Grossmann, Michael Gekle, Barbara Schreier

**Affiliations:** Julius-Bernstein-Institute of Physiology, Martin Luther University Halle-Wittenberg, Magdeburger Str. 6, 06110 Halle (Saale), Germany

**Keywords:** miR-221/222, Cardiomyocytes, Remodeling, L-type Ca^2+^ channel

## Abstract

**Background:**

Cardiovascular disease is the leading cause of death worldwide. Cardiac electrical remodeling including altered ion channel expression and imbalance of calcium homeostasis can have detrimental effects on cardiac function. While it has been extensively reported that miR-221/222 are involved in structural remodeling, their role in electrical remodeling still has to be evaluated. We previously reported that subunits of the L-type Ca^2+^ channel (LTCC) are direct targets of miR-221/222. Furthermore, HL-1 cells transfected with miR-221 or -222 mimics showed a reduction in LTCC current density while the voltage-dependence of activation was not altered. The aim of the present study was to determine the influence of miR-221/222 on cardiomyocyte calcium handling and function.

**Results:**

Transient transfection of HL-1 cells with miR-221/222 mimics led to slower depolarization-dependent Ca^2+^ entry and increased proportion of non-responding cells. Angiotensin II-induced Ca^2+^ release from the SR was not affected by miR-221/222. In miR-222-transfected neonatal cardiomyocytes the isoprenaline-induced positive inotropic effect on the intracellular Ca^2+^ transient was lost and the positive chronotropic effect on spontaneous beating activity was strongly reduced. This could have severe consequences for cardiomyocytes and could lead to a reduced contractility and systolic dysfunction of the whole heart.

**Conclusions:**

This study adds a new role of miR-221/222 in cardiomyocytes by showing the impact on β-adrenergic regulation of LTCC function, calcium handling and beating frequency. Together with the previous report that miR-221/222 reduce GIRK1/4 function and LTCC current density, it expands our knowledge about the role of these miRs on cardiac ion channel regulation.

**Supplementary Information:**

The online version contains supplementary material available at 10.1186/s13578-021-00676-4.

## Background

Cardiac remodeling comprises all changes in molecular, cellular and interstitial composition of the heart and can lead to cardiac dysfunction, malignant arrhythmias, sudden cardiac arrest or heart failure [1]. More specific, electrical remodeling includes altered ion channel expression and imbalance of calcium homeostasis [2]. All together these molecular changes can lead to cardiac dysfunction, malignant arrhythmias, sudden cardiac arrest or heart failure [1]. Aberrant microRNA (miR) expression has been implicated in cardiac remodeling and disease [3]. miR-221/222 are among the miRNAs involved in these processes.

In the last decade our understanding of miR-221/222 in remodeling has deepened but whether these miRs are detrimental or beneficial is still unclear. MiR-221/222 are clustered miRNAs that share the same seed sequence [4]. They are ubiquitously expressed and conserved between e.g. mouse and human [3]. Although miR-221/222 do not belong to cardiac-enriched miRs like miR-1, miR-133, miR-208 and miR-499, they are also involved in cardiac pathology [3]. The role of miR-221/222 in cardiac remodeling is discussed highly controversial. Several studies show that miR-221/222 are linked to cardiac hypertrophy [5, 6]. Furthermore, studies investigating tissue or circulating miR-221/222 expression as potential biomarkers for cardiovascular disease suggested that miR-221/222 levels are associated with disease state [7–10], or cardiovascular risk [11]. In line with this, cardiac-specific overexpression of either miR-221 or miR-222 led to cardiac hypertrophy, cardiac fibrosis, apoptosis, reduced autophagy and development of heart failure (HF) in mice [12, 13]. However, other studies attribute a more cardioprotective role to miR-221/222 [6, 14–21]. Our group previously demonstrated that miR-221/222 are upregulated in the hearts of mice with either genetically (EGFR KO) or pharmacologically (angiotensin II) induced heart hypertrophy [22].

Among other findings, we demonstrated (i) that mRNA expression of the L-type Ca^2+^ channel (LTCC) subunits Cacna1c, Cacnb2 and Cacna2d1 was decreased in these hearts, (ii) that the 3’-UTRs of Cacna1c and Cacnb2 are direct targets of these miRNAs and (iii) that miR-221/222 mimics reduce LTCC current density in whole cell patch clamp recordings of HL-1 cells [22]. Thus, miR-221/222 contribute to cardiac electrical remodeling by reducing LTCC-mediated I_Ca,L_ current.

In the heart, the LTCC mediates action potential (AP) conduction as well as excitation–contraction coupling in cardiomyocytes and is therefore crucial for cardiac function. Accordingly, cardiac disease is often associated with LTCC remodeling [23–25]. The physiological adaptation of the I_Ca,L_ to biological needs is mediated by a high variety of signaling pathways and mechanisms that converge at modulating LTCC function. The most important modulators include β-adrenergic signaling, intracellular Ca^2+^ concentration and membrane voltage [26]. LTCC remodeling includes differential expression, function and regulation of this channel. Unresponsiveness to β-adrenergic stimulation is one hallmark of the failing heart [27] and may be caused in part by altered LTCC expression.

Since miR-221/222 directly regulate the LTCC and thereby the I_Ca,L_ [22], the aim of the present study was to determine the influence of miR-221/222 on cardiomyocyte calcium handling and function, including response to β-adrenergic stimulation and spontaneous beating frequency. Thereby miR-221/222 could affect physiological and pathophysiological adaptation of the cardiac output.

In this study we demonstrate that transfection of HL-1 cells with miR-221/222 led to slower depolarization-dependent Ca^2+^ entry and increased proportion of non-responding cells. AngII-induced Ca^2+^ release from the SR was not affected by miR-221/222. In miR-222-transfected neonatal cardiomyocytes (neoCM) the isoprenaline (ISO)-induced positive inotropic effect on the intracellular Ca^2+^ transient was lost and the positive chronotropic effect on spontaneous beating activity was strongly reduced. Thus, we here demonstrate a novel role of miR-221/222 in cardiomyocytes.

## Results

### ***LTCC-mediated Ca***^***2***+^***influx is slower in miR-transfected HL-1 cells***

**Fig. 1:**
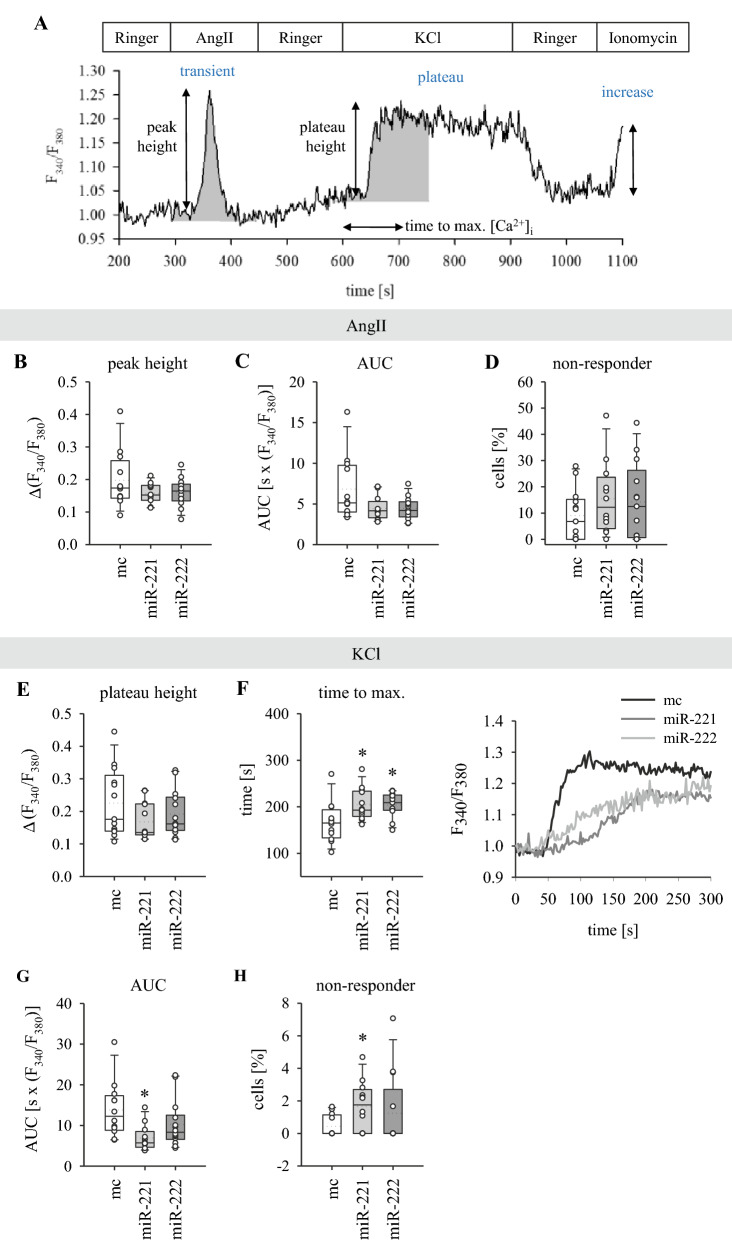
LTCC-mediated increase in cytosolic [Ca^2+^] is slower in miR-221/222-transfected HL-1 cells. **A** Scheme of the applied superfusion protocol and typical response of a cell to 100 nM AngII, 25 mM KCl and 1 µM ionomycin (mimic control). Grey areas indicate areas used for calculation of area under the curve (AUC). **B**–**D** AngII elicits a transient increase in [Ca^2+^]_i_. Peak height, percentage of non-responding cells and AUC are not significantly altered compared to mimic control. **E**–**H** KCl leads to a baseline shift of [Ca^2+^]_i_ up to a plateau level. The baseline shift is reduced by trend by miR-221/222. The area under the curve is significantly reduced in miR-221-transfected cells and miR-221/222 both lead to a significantly slower increase in [Ca^2+^]_i_ up to the plateau level. The time to max. is also shown in representative tracings (**F**, right side). The proportion of non-responding cells is significantly increased in miR-221-transfected cells. Straight line indicates median, dotted line indicates mean, *p  <  0.05, N  =  13–14 experiments per group (single data points), n  =  27–115 cells per experiment. *mc* mimic control

We previously reported that subunits of the L-type Ca^2+^ channel are direct targets of miR-221/222. To investigate if this regulation has an impact on cardiomyocyte calcium handling, Ca^2+^ homeostasis was analysed by ratiometric fluorescence microscopy in HL-1 cells. Two different mechanisms to increase cytosolic Ca^2+^ concentrations were tested: (1) a depolarization-independent increase mediated by AngII, followed by (2) a depolarization-dependent increase due to elevated extracellular K^+^ concentration. The sequence of stimulation and typical cellular reactions are depicted in Fig. 1A. AngII was used to investigate depolarization-independent effects of miR-221/222 on intracellular calcium release. Application of AngII evokes a transient increase in intracellular calcium concentration via an IP3/SR-dependent mechanism [28]. Superfusion with 25 mM KCl leads to cell membrane depolarization and subsequent LTCC activation causing a constantly increasing intracellular Ca^2+^ concentration up to a plateau level (baseline shift). To discriminate between dead cells and cells not responding to the respective stimuli, the calcium ionophore ionomycin was added to the superfusion at the end of each experiment. Living cells that do not respond to AngII and/or KCl still react to ionomycin with an increase in cytosolic Ca^2+^ concentrations. Dead cells on the other hand no longer have an intact plasma membrane, leading to an early accumulation of calcium in the cytosol and therefore no pronounced response upon ionomycin application.

Transfection of HL-1 cells with miR-221 or -222 has no impact on the AngII-induced calcium transient (Fig. 1B, C), neither on peak height [mimic control (mc): 0.20  ±  0.03; miR-221: 0.16  ±  0.01; miR-222: 0.16  ±  0.01 Δ(F_340_/F_380_)], nor on the area under the curve [mc: 6.84  ±  1.12; miR-221: 4.45  ±  0.41; miR-222: 4.48  ±  0.38 (F_340_/F_380_)s]. Furthermore, the percentage of cells not responding to AngII is not affected by miR-221/222 (Fig. 1D; mc: 9.06  ±  2.71%; miR-221: 15.43  ±  3.96%; miR-222: 14.25  ±  4.10%). Thus, miR-221/222 do not interfere with AngII-mediated depolarization-independent calcium signalling in HL-1 cells.

In contrast to this, LTCC-dependent calcium signalling is altered by miR-221/222. The KCl-induced baseline shift [Fig. 1E; mc: 0.23  ±  0.03; miR-221: 0.17  ±  0.02; miR-222: 0.19  ±  0.02 Δ(F_340_/F_380_)] is decreased by trend by miR-221 and -222. The area under the curve [Fig. 1G; mc: 13.82  ±  1.98; miR-221: 6.94  ±  0.90; miR-222: 10.28  ±  1.52 (F_340_/F_380_)s] is significantly reduced in miR-221-transfected cells and the time needed to reach maximum plateau Ca^2+^ levels is significantly prolonged by both miRs compared to control (Fig. 1F; mc: 166.80  ±  12.65 s; miR-221: 204.59  ±  9.37 s; miR-222: 204.22  ±  7.27 s). Additionally, miR-221 significantly increased the proportion of non-responding cells (Fig. 1H; mc: 0.44  ±  0.19%; miR-221: 1.66  ±  0.44%; miR-222: 1.25  ±  0.62%). These results show that miR-221/222 reduce depolarization-dependent Ca^2+^ influx in HL-1 cells.

**Fig. 2:**
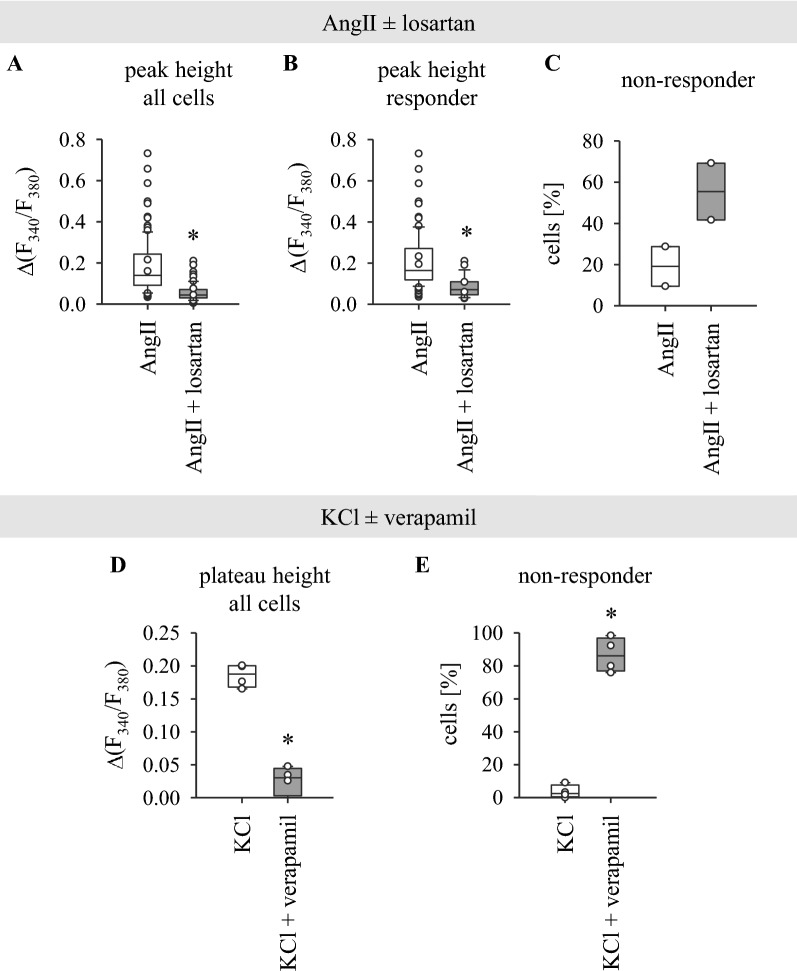
AngII- and KCl-induced changes in [Ca^2+^]_i_ are mediated by AT1R or LTCC, respectively. **A**–**C** AT1R inhibitor losartan significantly reduces the AngII-induced calcium transient in HL-1 cells. Cells were superfused either with 100 nM AngII (control) or with AngII plus 10 µM losartan. **A** Calcium transient peak of all cells (responding and non-responding cells combined) is reduced in losartan-treated cells. **B** Losartan also leads to diminished calcium transients in responding cells. **C** The proportion of non-responding cells is increased by losartan. Control: N  =  2, n  =  42–66 cells, losartan: N  =  2, n  =  24–39 cells (single data points). **D**, **E** LTCC blocker verapamil significantly reduces intracellular calcium increase in response to 25 mM KCl in HL-1 cells. Cells were superfused either with KCl (control) or with KCl plus verapamil. The baseline shift upon KCl superfusion is significantly reduced in verapamil-treated cells (**D**). **F** The dramatically reduced response when analysing all cells in this setting is in part due to an increase of non-responding cells. Concentration of verapamil: 20 µM, straight line indicates median, dotted line indicates mean, *p  <  0.05, N  =  4 (single data points), n  =  54–78 cells each

To verify that AngII- and KCl-induced calcium signals are indeed mediated by AT1R and LTCC, respectively, experiments with appropriate inhibitors were performed in untransfected HL-1 cells (Fig. 2). AT1R inhibitor losartan significantly reduces the AngII-induced calcium transient in comparison to control-treated cells (Fig. 2). Due to an increased population of cells that are no longer responding to AngII upon losartan application (Fig. 2C; losartan: 55.45  ±  13.78% vs control: 19.16  ±  9.63%), the overall peak height of all cells [Fig. 2A; losartan: 0.06  ±  0.02 vs control: 0.19  ±  0.03 Δ(F_340_/F_380_)] is even more reduced than that of the population of responding cells [Fig. 2B; losartan: 0.08  ±  0.004 vs control: 0.21  ±  0.001 Δ(F_340_/F_380_)]. LTCC blocker verapamil prevents the KCl-induced intracellular calcium increase by enlarging the population of non-responding cells (Fig. 2E; verapamil: 86.67  ±  5.25% vs control: 3.53  ±  1.97%). In line with this, when considering the overall response of all cells, the plateau height is dramatically reduced in verapamil-treated cells [Fig. 2D; verapamil: 0.03  ±  0.002 vs control: 0.18  ±  0.007 Δ(F_340_/F_380_)]. Both losartan and verapamil diminish the response elicited under control conditions. Thus, the observed cellular calcium response to AngII and KCl is indeed mediated by AT1R and LTCC, respectively.

In conclusion, the results of calcium measurements in HL-1 cells indicate that miR-221/222 do not influence the IP3/SR-mediated cytosolic calcium increase but have an effect on the depolarization-dependent (e.g., action potential-dependent) intracellular calcium increase in cardiomyocytes. Thus, downregulation of LTCCs by miR-221/222 may have an effect on depolarization kinetics in cardiomyocytes.

### Basal calcium handling is not affected by miR-221/222 in neoCM

**Fig. 3:**
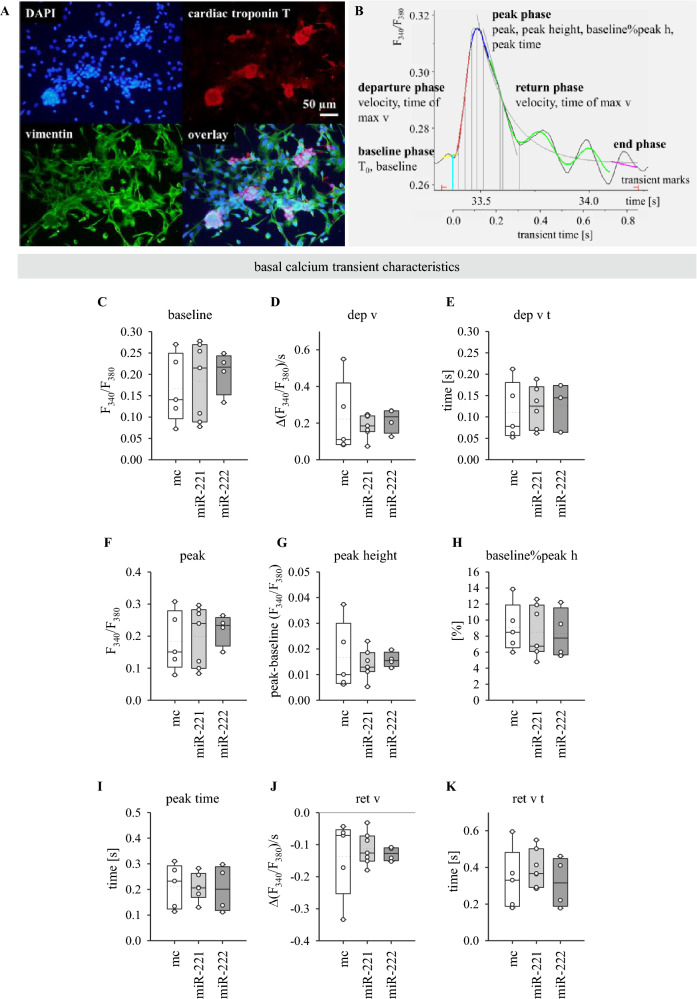
Calcium transient characteristics under control conditions are not affected by miR-221/222 in neoCM. **A** NeoCM form cell clusters with cardiac fibroblasts in culture. Immunofluorescence staining of ventricular neoCM after 6 days in culture. DAPI: nuclei, cardiac troponin T: cardiomyocytes, vimentin: fibroblasts. Scale bar indicates 50 µm. **B** Overview of most important monotonic transient analysis parameters calculated by IonWizard, sorted by the different phases of the transient. **C**–**K** Spontaneous or electrically evoked calcium transients were measured in monolayers under control conditions for 2 min. Monotonic transient analysis was performed to evaluate transient parameters for the baseline (**C**), departure (**D**, **E**), peak (**F**–**I**) and return phases (**J**, **K**) of the calcium transients. There is no significant difference between miR-221/222 and mimic control (mc). Straight line indicates median, dotted line indicates mean, mc: N  =  4–5, miR-221: N  =  6–7, miR-222: N  =  4

To analyse a possible effect of miR-221/222 on spontaneous calcium transients and beating activity, the next experiments were performed with murine neoCM. In our hands HL-1 cells do not show spontaneous beating activity. They also do not react to ISO, probably due to the fact that they are cultured with noradrenaline. In contrast, neoCM form functional spontaneously beating cell clusters after a few days in culture. Figure 3A shows that these clusters do not only contain CMs but also cardiac fibroblasts. This direct co-culture imitates the physiological in vivo situation more closely.

To investigate whether miR-221/222 influence basal calcium handling in neoCM, calcium transients were measured under control conditions for 2 min. Both spontaneous calcium transients and electrically evoked transients were recorded. Monotonic transient analysis was performed using IonWizard software (IonOptix) to evaluate transient parameters for the baseline, departure, peak and return phases of the calcium transients (Fig. 3B). The baseline is defined as the fluorescence ratio at the beginning of the transient (T_0_). The departure velocity (dep v) describes the speed of the increase in intracellular Ca^2+^. It is calculated as the maximum rate of change in fluorescence ratio during the departure phase. The time to maximum departure velocity (dep v t) is defined as the time interval between T_0_ and the maximum dep v. The peak is the maximum fluorescence ratio during the transient. The peak height is the difference between baseline and peak, i.e., the amplitude of the transient. The parameter baseline%peak height describes the peak amplitude as percent change of basal calcium levels in the cells. The parameters for the return phase, return velocity (ret v) and time of maximum return velocity (ret v t), are calculated like the ones for the departure phase.

Under unstimulated conditions there was no statistically significant difference in calcium transient parameters between miR-221/222 and mimic control (Fig. 3C–K). The high variability observed especially in the mc group is due to differences between the neonatal cardiomyocyte preparations. Two out of five monolayers showed higher values than the rest especially for departure velocity, peak height and return velocity. However, they also responded to ISO like the other monolayers.

### The effect of ISO on calcium transients is lost in miR-222-transfected neoCM

**Fig. 4:**
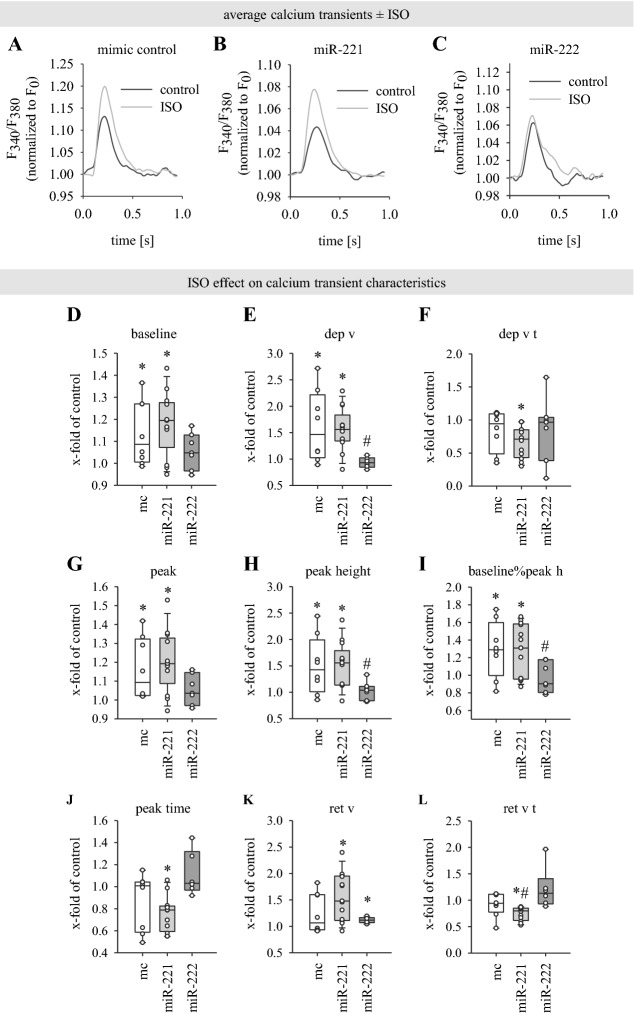
The positive inotropic effect of ISO on [Ca^2+^]_i_ is lost in miR-222-transfected neoCM monolayers. **A**–**C** Average calcium transients show differential response to 10 µM ISO in miR-222-transfected monolayers. Exemplary average transients are obtained from paced transients only (at least 5 transients) from one measurement each. Transients are representative for ISO effect on transient amplitude characteristics, not for baseline and kinetic parameters since data were normalized to fluorescence ratio at timepoint 0 (“F_0_”) and kinetic parameters vary slightly between measurements within one group. **D**–**L** Spontaneous or electrically evoked calcium transients were measured in monolayers under control conditions for 2 min, and after ISO addition after further 2 and 6 min for 3 min each. Following monotonic transient analysis, the ISO effect was calculated as ratio of mean transient parameter under ISO exposure divided by mean transient parameter under control conditions for each monolayer. Therefore, data are shown as x-fold of control. For units of the shown parameters and absolute values under control conditions see Fig. 3. ISO effect is shown for parameters describing baseline (**D**), departure (**E**, **F**), peak (**G**–**J**) and return phase (**K**, **L**) of calcium transients. While ISO accelerates and enhances increase in cytosolic [Ca^2+^] in neoCM transfected with mimic control and miR-221, this effect is lost in miR-222-transfected monolayers. Data are displayed as mean  ±  sem. Paired t test was used to directly compare the calcium transient parameters under control condition and after ISO application for each monolayer.  *p  <  0.05 ISO effect: ISO compared to respective control (no ISO  =  1), ^#^p < 0.05 ISO effect in miR-transfected cells compared to ISO effect in mc-transfected cells. Straight line indicates median, dotted line indicates mean, mc: N  =  8, miR-221: N  =  12–13, miR-222: N  =  6–7

To compare cellular responses in miR-221-, miR-222- or mimic control-transfected neoCM monolayers, cells were stimulated with ISO to elicit an activating effect on LTCC and calcium-induced calcium release (CICR). Figure 4 shows the ISO effect in mimic control-, miR-221- and miR-222-transfected monolayers. Due to variability of calcium transients between different neoCM preparations, transient characteristics under ISO were directly compared to the respective control. For this, the ISO effect was calculated as follows: mean transient parameter (ISO)/mean transient parameter (control). Therefore, data are represented as x-fold change of control.

The results show that mimic control-transfected neoCM monolayers react to ISO (Fig. 4A). Exposure to 10 µM ISO for in total 5–9 min significantly increased baseline fluorescence (Fig. 4D). As expected, departure velocity of the calcium transients was elevated as well (Fig. 4E). The time of maximal departure velocity (dep v t, Fig. 4F) was slightly, but not significantly reduced. Peak and peak height of the calcium transients were elevated as well (Fig. 4G, H). As mentioned above, the parameter baseline%peak height describes the peak amplitude as percent change of basal calcium levels in the cells. In contrast to peak height, this parameter provides higher comparability of the ISO effect between samples. Accordingly, the ISO effect on baseline%peak height is highly significant (Fig. 4I; 1.29  ±  0.11; p  =  0.0292). While ISO slightly reduced time to peak, return velocity and time to reach maximal return velocity (Fig. 4J–L), these results did not reach statistical significance. All in all, there is a measurable ISO effect on departure velocity and peak amplitude in spontaneous or pacing-evoked calcium transients of mimic control-transfected neoCM monolayers. ISO application led to differential results in miR-transfected cells. While ISO evoked a response similar to mc in miR-221-transfected cells (Fig. 4B), virtually no effect of ISO could be detected in miR-222-transfected cells (Fig. 4C). Apart from return velocity, which is minimally increased, no parameter is significantly changed upon ISO addition in miR-222-transfected neoCM. Furthermore, there is a significant difference in the ISO effect between mc and miR-222 for the departure velocity, peak height and baseline%peak height. Thus, the ISO effect observed in mc-transfected cells is lost in miR-222-transfected cells.

### The ISO-induced increase in spontaneous contraction frequency is abolished by miR-222

**Fig. 5:**
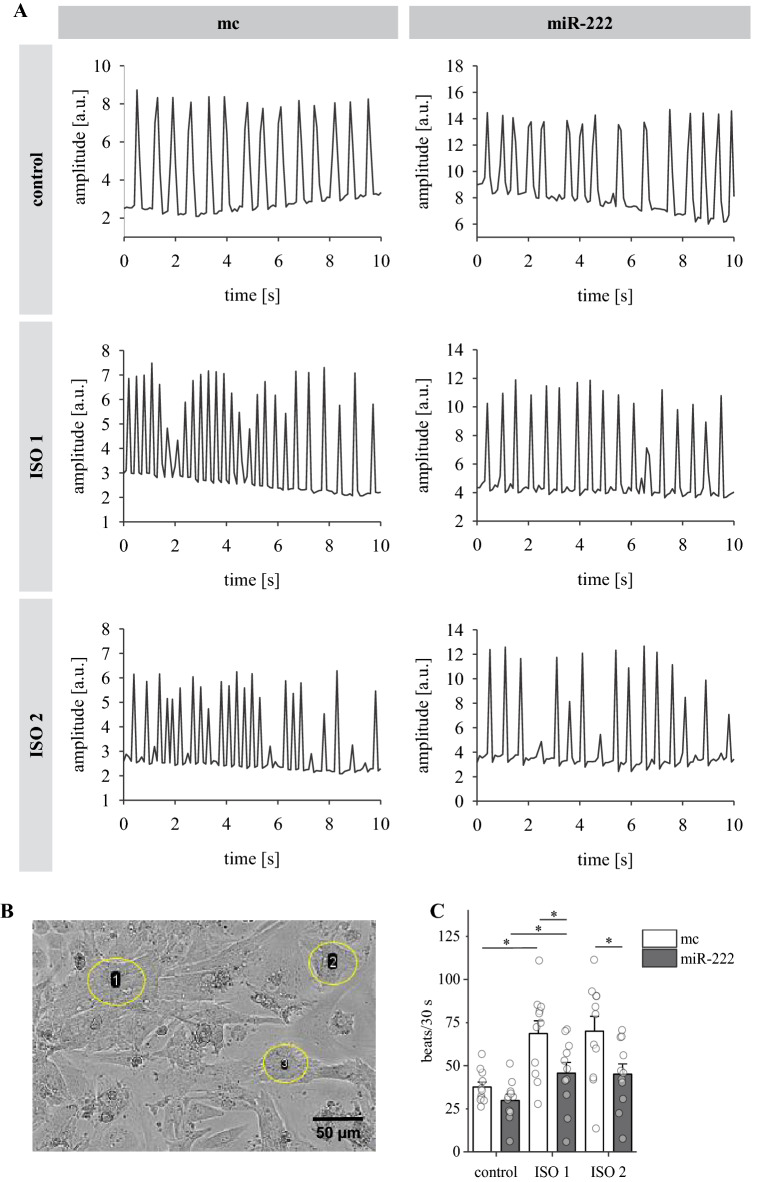
The positive chronotropic effect of ISO is significantly reduced in miR-222-transfected neoCM. Spontaneous contraction activity was recorded at 37°C for 30 s each. ISO1 was measured 2 min after buffer change to ISO and ISO2 was measured 1.5 min after ISO1 (for exact timeline, see Methods section). **A** Representative contraction tracings are shown for mimic control and miR-222 for 10 s each (a.u.: arbitrary units). Corresponding movies and ROI overviews can be found in the Additional material (Additional files 4, 5, 6, 7, 8, 9, 10, 11, 12, 13, 14, 15, 16, 17). **B** Exemplary section of an ROI overview image of a neoCM monolayer (mc). Shown are three manually placed ROIs (yellow circles), scale bar: 50 µm. **C** While the number of spontaneous contractions was almost doubled in mc-transfected cells upon ISO application, this ISO effect is significantly reduced in miR-222-transfected cells. There is no difference between both ISO time points within each group. Data include 3 independent experiments, N  =  11 petri dishes, n  =  5–37 cells or cell clusters, depending on size of clusters or confluence of monolayer. Data are displayed as mean  ±  sem, single data points represent the mean from each petri dish, *p  <  0.05 respective to mc or corresponding control conditions as indicated

After we established an impact of miR-221/222 on calcium entry and calcium transients due to depolarization or β-adrenergic stimulation, we wanted to know if miR-221/222 also affect cardiomyocyte beating frequency. Since miR-221 had no effect on calcium transients in neoCM, spontaneous contractions were only measured in miR-222-transfected neoCM and compared to mimic control. Occurrence of spontaneous beating was recorded for 30 s each under control conditions and after 10 µM ISO addition. As expected, ISO induces an increase in spontaneous contraction frequency (positive chronotropic effect) in mimic control-transfected cells (Fig. 5, for analysed movies see Additional files 12, 13, 14). At 37°C the basal spontaneous beating frequency is 37.4  ±  3.3 beats per 30 s and it is increased to 71.7  ±  8.5 beats after addition of ISO (ISO1; Fig. 5C). There was no significant difference in spontaneous contraction frequency between mimic control and miR-222-treated neoCM under baseline conditions. However, the increase in spontaneous contraction frequency by ISO is strongly diminished in miR-222-transfected cells (Fig. 5, for analysed movies see Additional files 15, 16, 17). With miR-222, cells contracted 28.4  ±  4.0 times in 30 s and ISO addition led to an increase to only 40.8  ±  7.1 beats (ISO1). Since calcium measurements in HL-1 cells revealed that intracellular calcium increase was slowed in miR-transfected cells, the possibility of a delayed onset of ISO response was tested by including a second recording after ISO1 (ISO2). However, there was no change in beating frequency between both time points (ISO2: mc: 72.4  ±  10 beats/30 s vs. miR-222: 40.5  ±  7.1 beats/30 s).

## Discussion

### miR-221/222 impair LTCC-induced calcium increase but not intracellular calcium release

Measurements of cytosolic calcium in HL-1 cells clearly showed that miR-221/222 reduce LTCC-mediated calcium increase due to significantly prolonged time to reach plateau calcium levels (miR-221/222) as well as significantly increased proportion of non-responding cells (miR-221). It is a limitation of this study that we did not measure a pure LTCC effect. Rather, we measured a combined effect of LTCC and SR. However, since we previously showed that miR-221/222 directly target LTCC subunit mRNAs and reduce LTCC current density in HL-1 cells [22] and cardiomyocytes depend on the entry of extracellular calcium for excitation–contraction coupling, this experiment still shows an effect on the LTCC.

In contrast to the response to KCl, AngII-induced calcium transients were unaffected by miR-221/222. Since AngII elicits the observed transient increase in cytosolic calcium through release from intracellular stores [28], it can be used to estimate the influence of miR-221/222 on SR calcium load. While miR-221/222 impair LTCC-dependent calcium entry, handling of intracellular stores does not seem to be affected. This supports the notion that the miR-effect is mainly due to LTCC regulation. Because the effect of AngII on calcium release is independent of LTCC and there was no difference in response between miRs and control, we concluded that SR calcium load per se is not affected by miR-221/222. Of course, this is only an indirect measure and does not exclude an effect of miR-221/222 on the RyR. To study SR calcium load in more detail, RyR and SERCA2a function could be analysed in future experiments, using caffeine to activate RyR2 and thapsigargin to block SERCA2a.

All in all, miR-221/222 affect the depolarization-dependent LTCC-initiated intracellular calcium increase in HL-1 cells. This could influence action potential generation, propagation and excitation-contraction coupling in cardiomyocytes in the heart.

### miR-222 abolishes the ISO-induced inotropic effect on intracellular calcium transients in neoCM

As expected, ISO had a positive inotropic effect on intracellular calcium transients in neoCM. With miR-222, this ISO effect was completely gone. This is in good agreement with our previous results from luciferase assay [22], calcium measurements in HL-1 cells and also patch clamp measurements [22] showing that LTCC subunits are targets of miR-222.

But, in contrast to miR-222, there was no alteration of the ISO effect in miR-221 transfected neoCM. However, calcium measurements and also patch clamp in HL-1 cells showed an influence of miR-221 on LTCC-dependent calcium handling. This discrepancy can be explained by the different methods and thereby the different read-outs. Intracellular calcium in HL-1 cells was measured after application of KCl until a clear plateau phase was observed, for 300 s in total. In this case, there was maximum activation of LTCC channels. Similar to this, I_Ca,L_ current was also measured at maximum activation of LTCC channels, so the impact of miR-221/222 on peak calcium current was analyzed. In contrast to these measurements, in neoCM no maximum activation but rather the more dynamic calcium handling important for excitation–contraction coupling was analyzed. NeoCM were paced at 1 Hz if there was no spontaneous activity, so calcium cycling had to be much faster and there probably was no maximum activation. This suggests that the miR-221 effect might only be seen at maximum activation. However, since the results from neoCM dynamic calcium handling are functionally more relevant to the in vivo situation, miR-222 seems to be more important than miR-221 in regulating LTCC.

There are some limitations with calcium measurements in neoCM. Although a pre-plating step was added during isolation to reduce the number of non-cardiomyocytes, neoCM monolayers are not only cardiomyocytes [29] (see also Fig. 3A). Since neoCM need a few days after preparation to fully regain their properties and then have to be transiently transfected, measurements could be performed only after about seven days in culture. At this point, there was a considerable number of non-cardiomyocytes in the neoCM culture, even under culture conditions with only 1% FCS. So, it cannot be excluded that measured effects are also due to interaction of miR-transfected fibroblasts or endothelial cells with cardiomyocytes. Additionally, there is likely an underestimation of the miR effect because probably not all cells in the monolayer are transfected and cells are still proliferating after transfection, thereby further diluting the proportion of transfected cells and the observable miR effect. Also, a difference in the effects of miR-221/222 between neonatal and adult cardiomyocytes cannot be excluded.

### The ISO-induced increase in spontaneous contraction frequency is abolished by miR-222

NeoCM react to ISO not only with faster and higher calcium transients, they also increase their spontaneous beating activity, thus implementing a positive chronotropic response. MiR-222 did not only influence the effect of ISO on calcium transients but also on spontaneous beating frequency in neoCM. While basal spontaneous contraction activity was similar in control and miR-222-transfected neoCM, the response to ISO was significantly reduced. This suggests reduced β-adrenergic responsiveness in miR-222-transfected cells which is an important feature of CMs in HF [27]. Since miR-221 had no effect on the ISO response regarding the calcium transients, it was not further analysed. The effect of miR-222 is probably due to regulating LTCC expression, as it is known that verapamil blocks spontaneous calcium transients [30] and contractions (Fig. 5, Additional file 1: Figure S1; movies: Additional files 2, 3).

In contrast to calcium measurements, contractions were recorded in unstained neoCM monolayers with brightfield illumination, so there was no problem with dye loading or photobleaching. Increasing the bath temperature of the neoCMs from room temperature to 34–37°C improved their spontaneous beating activity. Previous calcium measurements were performed at room temperature and often there was no spontaneous activity so that calcium transients had to be evoked electrically. Again, there likely is an underestimation of the miR effect due to transfection of probably only a portion of cells and further proliferation of neoCMs until the measurement.

### Possible influence of reduced LTCC protein expression on calcium homeostasis in the heart

Since the LTCC is essential for action potential conduction and excitation-contraction coupling throughout the heart, a reduction in functional channel density could greatly impair cardiac function. MiR-221/222 transfection led to several alterations in calcium handling in HL-1 cells and neoCM that would impact the whole heart. First, under unstimulated conditions, KCl-induced Ca^2+^ entry was slowed and reduced in miR-transfected HL-1 cells compared to mimic control. This implies that basal LTCC activity is reduced in these cells, most likely due to posttranscriptional downregulation by miR-221/222. In the present study the previously reported direct interaction of miR-221/222 with Cacna1c mRNA, which encodes the Cav1.2 pore forming subunit, was associated with a reduction in channel function. Furthermore, the effect of miR-221/222 on L-type Ca^2+^ current (I_Ca,L_) density was analyzed by whole cell patch clamp recording [22]. It has been reported that HL-1 cells express several voltage-gated Ca^2+^ channels. Apart from LTCCs, of which Cav1.2 is higher expressed and mostly localized on the cell surface while Cav1.3 is lowly expressed and mostly located intracellularly, they also express T-type Ca^2+^ channels (TTCC) [31]. To ensure that the observed Ca^2+^ current was mediated by LTCC and not TTCC, I_Ca,L_ was elicited at −40 mV which is too low for TTCC activation [31]. TTCCs activate at around −70 mV [31]. Transfection with mimics for both miRs decreased I_Ca,L_ density in HL-1 cells significantly, however, the voltage-dependence of activation was not altered [22]. Thus, the downregulation of peak I_Ca,L_ seems to be mainly due to transcriptional regulation of LTCC subunit mRNAs by miR-221/222.

The second observation was that β-adrenergic stimulation with ISO resulted in a reduced calcium transient amplitude in neoCM monolayers transfected with miR-222 compared to mimic control. This suggests a reduced positive inotropic effect of ISO due to miR-222. Since contractility per se was not measured, the effect of miR-221/222 on inotropy can only be discussed insofar as the calcium transient amplitude is thought to correlate with force development [32]. However, an inadequate response to β-adrenergic stimulation impairs the ability of the heart to adapt the cardiac output to differing needs and thus impairs cardiac function. This is in line with the observation that the β-adrenergic reserve is reduced in HF [33].

This also fits well to the third observation, that β-adrenergic stimulation resulted in a reduced positive chronotropic response in miR-222-transfected neoCM compared to mimic control. Ventricular neoCM exhibit spontaneous beating activity because they still express HCN channels (I_f_), TTCC and Cav1.3 as well as other components of the Ca^2+^ clock [34]. They express less TTCC than LTCC and the t-tubules are still in development but CICR, RyR, SERCA and SR calcium buffering are present [34]. In the heart the positive chronotropic effect of ISO is mediated by sinoatrial node (SAN), atrioventricular node (AVN) and conduction within the myocardium.

Transferring these results to the whole heart could mean that either pacemaking frequency is still increased due to ISO but conduction to or within the myocardium is slowed or that pacemaking frequency itself cannot adapt to ISO. Since SAN and AVN cells express mainly Cav1.3 and not Cav1.2 and until now we do not know whether miR-221/222 target also Cav1.3, heart rate may not be altered under control conditions. However, the most problematic result would be that the Ca^2+^ entry and therefore the contractility of the working myocardium in atria and ventricles is reduced under β-adrenergic stimulation because they express mainly Cav1.2 (atria also Cav1.3). The reduced cardiac function would lead to an elevated β-adrenergic tone but together with the reduced responsiveness to catecholamines this initiates and reinforces a vicious circle that in the end leads to failing of the heart [27]. Further experiments are necessary to determine the role of miR-221/222 on contractility of neoCM.

That basal calcium transients as well as spontaneous beating frequency were not affected by the miRs could be explained by a certain buffering in LTCC biosynthesis as described by Rosati et al. [35]. If this was the case, LTCC expression may be reduced by miR-222, but would still be sufficient to ensure spontaneous activity under basal conditions. However, then the β-adrenergic reserve would be too low to allow for adequate increase in intracellular Ca^2+^ transient amplitude with ISO. In contrast to patch clamp and KCl where peak current and maximum Ca^2+^ entry were measured, spontaneous contractions under unstimulated conditions should not require maximal activation of all LTCCs present and thus even reduced LTCC expression should be sufficient. An alternative explanation would be that LTCC expression is reduced to a greater extent by miR-221/222 and that the fewer channels are in a more active state even under control conditions. In this case, ISO-induced PKA-dependent phosphorylation of LTCC could not increase LTCC activity and thus beating frequency any further.

### Role of miR-221/222 in cardiac disease

MiR-221/222 are frequently upregulated in cardiac disease [5, 7–10, 36, 37] and seem to be associated with cardiac remodeling. Two studies with cardiac-specific overexpression of miR-221 or miR-222 in mice demonstrated cardiac hypertrophy and HF following miR overexpression [12, 13]. This confirmed findings from other studies linking miR-221 and especially miR-222 to cardiac hypertrophy [5, 6]. Although cardiac hypertrophy is thought to arise due to increased Ca^2+^ entry or [Ca^2+^]_i_ [38], there is also evidence for the opposite situation. Mice with cardiac-specific heterozygous Cav1.2 deletion developed even more pronounced cardiac hypertrophy under β-adrenergic activation and reduced ventricular function than control mice [25]. In contrast to the two studies of Su et al. miR-221 and especially miR-222 have been shown to be more cardioprotective than detrimental, so the question arises whether a reduction of LTCC expression is beneficial. In fact, studies with LTCC inhibitors point in this direction. Benidipine inhibited cardiac hypertrophy and prevented HF in a mouse model with pressure overload [39] and diltiazem prevented disruption of SR Ca^2+^ homeostasis in mice with hypertrophic cardiomyopathy [40]. Whether miR-221/222 promote remodeling or protection may depend on the precise context of the respective cardiac disease. In the present study we could show that miR-221/222 impact the β-adrenergic response in cardiomyocytes by reducing LTCC function.

Nevertheless, it cannot be excluded that the observed miR-221/222 effect may also be partly mediated by the regulation of other putative targets. For example, RyR is also among the predicted targets of miR-221/222 and also downregulated in hearts of EGFR KO mice where miR-221/222 were upregulated [22]. Until now this putative target was not confirmed but its regulation would probably contribute to electrical remodeling, further reducing SR calcium load and thereby contractility.

### Differential effects of miR-221 and miR-222

It seems that miR-222 elicits a stronger effect on LTCC in neoCM than miR-221, which is therefore also detectable even when LTCC is not maximally activated. While miR-221 also reduced peak I_Ca,L_ and slowed KCl-induced increase in [Ca^2+^]_i_, it had no effect on the response to β-adrenergic stimulation, in contrast to miR-222. The β-subunit (Cacnb2) that enhances LTCC trafficking to the cell membrane is a confirmed target of miR-222 [41]. Loss of this subunit could, at least in part, explain the stronger effect of miR-222 on calcium handling in cardiomyocytes. It is also conceivable that miR-221 may additionally target regulators of I_Ca,L_, e.g., phosphatases, thereby contributing to preserved LTCC function. Differential targeting of miR-221/222 despite sharing the same seed sequence has been described before [42]. There is evidence that also the central and 3’-region of the miR may be important for target binding [42]. Additionally, miR-222 was lower expressed than miR-221 in hearts from WT and EGFR KO mice [22]. This suggests independent transcription [43] or posttranscriptional regulation of miR transcripts [44] which could further strengthen differential effects of miR-221 and -222.

## Conclusion

This study adds a new role of miR-221/222 in cardiomyocytes by showing the impact on β-adrenergic regulation of LTCC function, calcium handling and beating frequency. Together with the previous report that miR-221/222 reduce GIRK1/4 function and LTCC current density, it expands our knowledge about the role of these miRs on cardiac ion channel regulation. Future studies in neoCM over a prolonged time or in mouse models are necessary to uncover potential miR-221/222-induced compensatory mechanisms caused by reduced LTCC function and their effect on long-term cardiac remodeling and function.

## Materials and methods

More detailed descriptions of neoCM isolation, calcium measurements and contraction analysis can be found in the supplemental information (Additional file 1).

### Ethics statement

All mouse experiments described in this manuscript were performed according to the guidelines of the directive 2010/63/EU of the European Parliament on the protection of animals used for scientific purposes.

### Mouse models

Wildtype C57BL/6 J mice were kept in the facilities of the University of Halle-Wittenberg at a room temperature of 20  ±  1°C, with a 12 h/12 h light/dark cycle and were fed ad libitum with standard chow.

### Isolation and culture of neoCM and cardiac fibroblasts

A detailed description can be found in the supplemental information (Additional file 1). In brief, neoCM were isolated from wildtype C57BL/6 J newborn mice on postnatal day 0–2. Mice were sacrificed by decapitation and hearts were removed and placed in cold 0.9% NaCl solution. Whole hearts were digested in 10 min steps using digestion buffer (HBSS, 0.5 mg/ml trypsin, 20 µg/ml DNase II). Isolated cells were resuspended in neoCM medium (DMEM with 4.5 g/l glucose, 5% FCS, 20 µg/ml vitamin B_12_) supplemented with 25 µl/ml penicillin/streptomycin. After the initial seeding the FCS content was reduced to 1% to minimize fibroblast growth.

### Cell culture

All cells were cultured at 37°C and 5% CO_2_. HL-1 cells were maintained in Claycomb medium with the following supplements: 10% FCS, 2 mM L-glutamine, 100 µM noradrenaline, 100 U/ml penicillin and 100 µg/ml streptomycin. Cells were subcultured at confluency (once a week; 1/10) and medium was changed twice a week.

### Transient transfection with miR-221/222 mimics

For transient transfection of cells with miR-221/222 miRCURY LNA microRNA mimics (Qiagen, Venlo, Netherlands) without labels were used. HL-1 cells were transiently transfected with 30 nM of miRCURY LNA miR-221/222 mimics or mimic negative control using 5 µl lipofectamine 2000 (Thermo Fisher Scientific, Waltham, Massachusetts, USA) in 1.5 ml DMEM (Biochrom; without FCS) following manufacturer’s instructions. After 24 h the medium was changed and cells were kept on Claycomb medium with supplements for further 48 h. NeoCM were transiently transfected like HL-1 cells. After 24 h the medium was changed and cells were kept on DMEM with 4.5 g/l glucose, 20 µg/ml vitamin B_12_ and 5% FCS for further 48 h.

### Calcium imaging in HL-1 cells

Calcium homeostasis in HL-1 cells was measured by ratiometric fluorescence microscopy using Fura-2 AM. HL-1 cells were plated on FCS-coated custom-made glass coverslips for 24 h in Claycomb medium with supplements. MiR-transfected HL-1 cells were loaded with 4 µM Fura-2 AM (stock solution: 1 mM in DMSO) at 37°C for 30 min. During the experiment cells were superfused with pre-warmed control and test solutions (temperature when reaching the cells: 37°C). Ringer buffer (control solution) contained in mM: NaCl 122.5, KCl 5.4, MgCl_2_  ×  6 H_2_O 0.8, CaCl_2_  ×  2 H_2_O 1.2, NaH_2_PO_4_  ×  H_2_O 1.0, glucose 5.5, HEPES 10; pH was adjusted to 7.4 at 37°C. The measurement started with control solution for 200–300 s to get a stable baseline signal, followed by superfusion with 100 nM AngII for 150 s to observe a transient [Ca^2+^]_i_ increase. After this, control Ringer solution was applied for 150 s to wash out AngII. To depolarize the cell membrane and activate LTCCs, the next step included Ringer solution with 25 mM KCl (instead of 5 mM in control solution) for 300 s. In a last step, cells were superfused with 1 µM ionomycin which serves as a positive control to discriminate between non-responding and dead cells. To test whether AngII and 25 mM KCl elicit the expected specific effects, inhibitors of AngII receptor type 1 (AT1R) (losartan, 10 µM) and LTCC (verapamil, 20 µM) were applied in additional experiments. Images of Fura-2 fluorescence intensity at excitation wavelengths of 340 nm and 380 nm were obtained with VisiVIEW Imaging Software (Visitron Systems; exposure: 40 ms, bin: 2, sampling interval: 2 s, emission: 510 nm). Analysis was performed on single cell level by defining one region of interest per cell. 340/380 ratios were exported to Excel. Background fluorescence was subtracted. The reaction of a cell to a substance was defined as a response if the maximum value was higher than the mean plus three times the standard deviation of the control solution before substance application [e.g., maximum value (AngII)  >  mean (Ringer)  +  3  ×  standard deviation (Ringer)]. First, values from dead cells were eliminated due to lack of response to ionomycin. Then, responding and non-responding cells were defined. Response parameters, e.g., baseline shift, peak height, AUC (AngII: 140 s, KCl: first 150 s), were only obtained from responding cells unless indicated otherwise.

### Calcium measurement in neoCM

Calcium transients from spontaneous or electrically evoked activity in neoCM were obtained with the Myocyte Calcium and Contractility System (IonOptix, Westwood, Massachusetts, USA). NeoCM were measured 6–9 days after isolation. NeoCM monolayers were transiently transfected with miR mimics for 24 h as described above. Cells were loaded with 4 µM Fura-2 AM (stock solution: 1 mM in DMSO) at 37°C for 30 min. Each sample was measured three times at room temperature: after recording control signals for 2 min the buffer was changed to 10 µM isoprenaline (ISO) (2 min break) and response to ISO was measured for 3 min and after a 1 min break again for 3 min. If possible, spontaneous calcium transients were recorded. Otherwise, cells were paced at 1 Hz to evoke calcium transients (MyoPacer, 5–10 V per pulse, pulse duration maximum 4 ms). IonWizard 6.6 (IonOptix) was used for data acquisition at sampling frequencies of 100 Hz (average 4: 4 collected data points are averaged into one raw data point) or 250 Hz (average 1). Monotonic transient analysis was performed using IonWizard 6.6.

### Contraction analysis in neoCM

NeoCM were isolated as described above and cells from about one heart were plated onto one 35 mm petri dish. Monolayers were transfected with mimic control or miR-222 as described above and measured after about 7–8 days in culture. Before the recording cells were washed with Ringer buffer once. Cells were then placed into a pre-heated Ibidi chamber (Ibidi heating system 1; plate temperature: 38°C, lid: 42°C; Ibidi, Gräfelfing, Germany) and allowed to acclimatize for 1 min. Contraction recordings were performed as follows: acclimatization of the cells in Ringer for 60 s, movie 1 (control) 30 s, buffer change to 10 µM ISO and acclimatization 150 s, movie 2 (ISO 1) 30 s, break 90 s, movie 3 (ISO 2) 30 s. Movies were analyzed using Myocyter [45] with the following parameters: “detection”: 10, “% of max recognized as beat”: 20.

### Statistical analysis

Data are presented as mean  ±  standard error of mean. Student’s t test, Mann–Whitney rank sum test or Wilcoxon signed rank test were used as applicable according to pre-test data analysis by SigmaPlot 12.5. Tests were unpaired and two-tailed unless stated otherwise in the text. Grubbs tests were performed to identify outliers. A p value  <  0.05 was considered statistically significant. Graphics were created using Sigmaplot 12.5 and Origin 2018.

## Supplementary Information

**Additional file 1.** Additional figure 1 (Contraction-inhibiting effect of verapamil depends on neoCM cluster size), list of supplemental movies and images, supplementary methods. **Figure S1**. Spontaneous beating activity at room temperature was measured under control conditions (for ca. 54 s) and at two successive time periods after solution change to 10 μM verapamil (verapamil 1: ca. 54 s movie, verapamil 2: ca. 58 s movie). Beats were analysed using Myocyter Image J plugin. **A** Cluster-specific beating activity. **B** Statistical analysis. N = 1, n = 9 cluster, paired t-test, *p < 0.05. The corresponding movies can be found in the supplemental material.

**Additional file 2: Figure S2.** ROI overview: Untransfected neoCM spontaneous contraction control (for quantification see Additional file 1: Figure S1).

**Additional file 3**: **Figure S3.** ROI overview: Untransfected neoCM spontaneous contraction verapamil (for quantification see Additional file 1: Figure S1).

**Additional file 4**: **Figure S4.** ROI overview: mc-transfected neoCM spontaneous contraction control (for corresponding tracings of ROI 3 see Fig. 5A).

**Additional file 5**: **Figure S5.** ROI overview: mc-transfected neoCM spontaneous contraction ISO 1 (for corresponding tracings of ROI 3 see Fig. 5A).

**Additional file 6: Figure S6.** ROI overview: mc-transfected neoCM spontaneous contraction ISO 2 (for corresponding tracings of ROI 3 see Fig. 5A).

**Additional file 7: Figure S7.** ROI overview: miR-222-transfected neoCM spontaneous contraction control (for corresponding tracings of ROI 1 see Fig. 5A).

**Additional file 8: Figure S8.** ROI overview: miR-222-transfected neoCM spontaneous contraction ISO 1 (for corresponding tracings of ROI 1 see Fig. 5A).

**Additional file 9: Figure S9.** ROI overview: miR-222-transfected neoCM spontaneous contraction ISO 2 (for corresponding tracings of ROI 1 see Fig. 5A).

**Additional file 10: Movie S1.** Untransfected neoCM spontaneous contraction control (for quantification see Additional file 1: Figure S1).

**Additional file 11: Movie S2.** Untransfected neoCM spontaneous contraction verapamil (for quantification see Additional file 1: Figure S1).

**Additional file 12: Movie S3.** mc-transfected neoCM spontaneous contraction control (for corresponding tracings of ROI 3 see Fig. 5A).

**Additional file 13: Movie S4.** mc-transfected neoCM spontaneous contraction ISO 1 (for corresponding tracings of ROI 3 see Fig. 5A).

**Additional file 14: Movie S5.** mc-transfected neoCM spontaneous contraction ISO 2 (for corresponding tracings of ROI 3 see Fig. 5A).

**Additional file 15: Movie S6.** miR-222-transfected neoCM spontaneous contraction control (for corresponding tracings of ROI 1 see Fig. 5A).

**Additional file 16: Movie S7.** miR-222-transfected neoCM spontaneous contraction ISO 1 (for corresponding tracings of ROI 1 see Fig. 5A).

**Additional file 17: Movie S8.** miR-222-transfected neoCM spontaneous contraction ISO 2 (for corresponding tracings of ROI 1 see Fig. 5A).

## Data Availability

Derived data supporting the findings of this study are available from the corresponding author MK on request.

## Abbreviations

[Ca^2^^+^]_i_: Intracellular calcium ion concentration
AngII: Angiotensin II
AT1R: Angiotensin II receptor type 1
a.u.: Arbitrary units
AUC: Area under the curve
AVP: Atrioventricular node
baseline%peak h: Peak height as percentage of baseline
Cacna1c: Calcium channel gene, voltage-dependent, L type, alpha 1C subunit
Cacna2d1: Calcium channel gene, voltage-dependent, alpha2/delta subunit 1
Cacnb2: Calcium channel gene, voltage-dependent, beta 2 subunit
cAMP: Cyclic adenosine monophosphate
Cav1.2: L-type Ca^2^^+^ channel pore-forming subunit alpha 1C
Cav1.3: L-type Ca^2^^+^ channel pore-forming subunit alpha 1D
DAPI: 4′,6-Diamidino-2-phenylindole
dep v (t): (Time of) maximal departure velocity
EGFR: Epidermal growth factor receptor
F_340_/F_380_: Ratio of fluorescence intensities at 340 nm and 380 nm excitation wavelengths
Fps: Frames per second
HCN: Hyperpolarization-activated cyclic nucleotide–gated channel
HF: Heart failure
I_Ca,L_: L-type Ca^2^^+^ current
IP3: Inositol 1,4,5-trisphosphate
ISO: Isoprenaline
KCl: Potassium chloride
KO: Knockout
LTCC: L-type Ca^2^^+^ channel
mc: Mimic control
miR: MicroRNA
neoCM: Neonatal cardiomyocytes
peak h: Peak height
peak t: Peak time (time to peak)
ret v (t): (Time of) maximal return velocity
ROI: Region of interest
SAN: Sinoatrial node
SERCA: Sarco/endoplasmic reticulum Ca^2^^+^ ATPase
SR: Sarcoplasmic reticulum
TTCC: T-type Ca^2^^+^ channel
WT: Wildtype
β-AR: β-Adrenergic receptor
