# Supplementary material for: Influence of miR-221/222 on cardiomyocyte calcium handling and function
Source: Cell Biosci. 2021 Aug 17;11:160. doi: 10.1186/s13578-021-00676-4 (PMC8369661; doi:10.1186/s13578-021-00676-4)
Supplement: Supplementary file 1 — Additional file 1. Additional figure 1 (Contraction-inhibiting effect of verapamil depends on neoCM cluster size), list of supplemental movies and images, supplementary methods. Figure S1. Spontaneous beating activity at room temperature was measured under control conditions (for ca. 54 s) and at two successive time periods after solution change to 10 μM verapamil (verapamil 1: ca. 54 s movie, verapamil 2: ca. 58 s movie). Beats were analysed using Myocyter Image J plugin. A Cluster-specific beating activity. B Statistical analysis. N = 1, n = 9 cluster, paired t-test, *p < 0.05. The corresponding movies can be found in the supplemental material. [file 13578_2021_676_MOESM1_ESM.docx]

# Supplementary information

**Influence of miR-221/222 on cardiomyocyte calcium handling and function**

**Authors & affiliations**

Maria Knyrim^1$^, Sindy Rabe^1^, Claudia Grossmann^1^, Michael Gekle^1^, Barbara Schreier^1^

^1^Julius-Bernstein-Institute of Physiology, Martin Luther University Halle-Wittenberg, Magdeburger Str. 6, 06110 Halle/Saale, Germany

**Author ORCIDs**

Maria Knyrim 0000-0003-4211-2361

Sindy Rabe no ORCID Number available

Michael Gekle 0000-0002-1581-8767

Claudia Grossmann 0000-0003-2026-1980

Barbara Schreier 0000-0002-3087-6439

^$^**Corresponding Author:**

Maria Knyrim

maria.knyrim@medizin.uni-halle.de

## Additional figure S1

**Figure S1. Contraction-inhibiting effect of verapamil depends on neoCM cluster size**. Spontaneous beating activity at room temperature was measured under control conditions (for ca. 54 s) and at two successive time periods after solution change to 10 µM verapamil (verapamil 1: ca. 54 s movie, verapamil 2: ca. 58 s movie). Beats were analysed using Myocyter Image J plugin. **A** Cluster-specific beating activity. **B** Statistical analysis. N=1, n=9 cluster, paired t-test, * indicates p < 0.05. The corresponding movies can be found in the supplemental material.

## List of supplemental movies and images

Movies from spontaneous contraction recordings under control conditions and after application of ISO or verapamil can be found in the supplemental material. Attached images are overviews of manual ROIs used for analysis with the Image J plugin Myocyter. An overview of the file names is given below. For the corresponding movies see Additional files 10 to 17. Scale bars are provided in the ROI overview images.

**untransfected neoCM spontaneous contraction verapamil (for quantification see Additional figure 1):**

Additional file 2_neoCM control.jpg

Additional file 3_neoCM verapamil.jpg

Additional file 10_neoCM control.mp4

Additional file 11_neoCM verapamil.mp4

**mc-transfected neoCM spontaneous contraction ISO (for corresponding tracings of ROI 3 see Fig. 5A):**

Additional file 4_neoCM mc control.jpg

Additional file 5_neoCM mc ISO 1.jpg

Additional file 6_neoCM mc ISO 2.jpg

Additional file 12_neoCM mc control.mp4

Additional file 13_neoCM mc ISO 1.mp4

Additional file 14_neoCM mc ISO 2.mp4

**miR-222-transfected neoCM spontaneous contraction ISO (for corresponding tracings of ROI 1 see Fig. 5A):**

Additional file 7_neoCM miR-222 control.jpg

Additional file 8_neoCM miR-222 ISO 1.jpg

Additional file 9_neoCM miR-222 ISO 2.jpg

Additional file 15_neoCM miR-222 control.mp4

Additional file 16_neoCM miR-222 ISO 1.mp4

Additional file 17_neoCM miR-222 ISO 2.mp4

## Supplementary methods

All materials, chemicals and reagents were purchased from Sigma Aldrich (Munich, Germany) unless stated otherwise.

**Isolation and culture of neonatal mouse cardiomyocytes and cardiac fibroblasts**

NeoCM were isolated from wildtype C57BL/6J newborn mice on postnatal day 0-2. Mice were sacrificed by decapitation and hearts were removed and placed in cold 0.9% NaCl solution. Hearts from all littermates were pooled. Connective tissue was removed from the hearts. Whole hearts were transferred into 5 ml digestion buffer (HBSS, 0.5 mg/ml trypsin, 20 µg/ml DNase II) and minced into small pieces. Digestion was performed in 6-8 successive 10 min steps in 25 ml beakers at room temperature and constant stirring at 300 rpm. Generally, digestion steps were performed until all tissue pieces were digested. After 10 minutes of digestion the undigested tissue was allowed to settle down to the bottom of the beaker and the supernatant containing single cells was transferred to a 15 ml tube with 6 ml FCS (for trypsin inactivation; Biochrom, Berlin, Germany). New digestion buffer was added to the still undigested tissue and stirred again for 10 min. After each digestion step, the supernatant/FCS mixture was centrifuged at 1000 rpm for 5 min at room temperature. The supernatant was removed and the cell pellet resuspended in neoCM medium (DMEM with 4.5 g/l glucose, 5% FCS, 20 µg/ml vitamin B_12_) supplemented with 25 µl/ml penicillin/streptomycin. To remove most of the non-cardiomyocytes (mainly fibroblasts) from the cardiomyocyte culture, a pre-plating step was included. For this, cells were incubated for 1-2 h on untreated 50 mm Petri dishes at 37°C. In contrast to the cardiomyocytes, fibroblasts adhere to the untreated surface and the medium containing mainly cardiomyocytes could be removed and was centrifuged again. The cell pellet was resuspended in neoCM medium without antibiotics and the cells were plated onto fibronectin-1-coated glass cover slides in 50 mm Petri dishes (about two hearts per Petri dish). The next day the medium was changed to remove dead cells. After the initial seeding, the FCS content was reduced to 1% to minimize fibroblast growth.

**Immunofluorescence**

Freshly isolated ventricular neoCM were plated on fibronectin-1-coated Multitest slides (Dunn, Asbach, Germany) and incubated for 24 h. Cells were fixated with 4% paraformaldehyde for 15 min at room temperature. After three washing steps with 1xPBS for 5 min each, cells were permeabilized using 1xPBS containing 1% TritonX-100 for 30 min at room temperature. This was followed by two washing steps: 10 min with 1xPBS with 1%SDS and 100 mM glycine and 10 min with 1xPBS with 100 mM glycine at room temperature. Cells were incubated with 1xPBS containing 10% goat serum, 10% donkey serum, 1% BSA for 30 min to block unspecific antibody binding sites and washed once with 1xPBS. Primary antibody solution was added and incubated overnight at 4°C. The next day, after three 5 min washing steps with 1xPBS, cells were incubated with secondary antibody solution for 45 min at room temperature in the dark. Afterwards, cells were again washed three times with 1xPBS for 5 min. To stain nuclei, DAPI working solution was added and cells were incubated for 2-15 min. After three further washing steps with 1xPBS slides were mounted with Dako fluorescence mounting medium (Agilent, Santa Clara, USA) and coverslips were added. The slides were incubated at 4°C overnight to dry. Background fluorescence was checked in samples treated with only secondary antibodies. Immunofluorescence was analysed using the fluorescence microscope BioZero BZ-8100 (Keyence, Osaka, Japan).

Primary antibodies: mouse anti-cTnT: dilution 1:300 (ab10214, Abcam, Cambridge, UK), rabbit anti-vimentin: 1:100 (#5741, Cell Signaling Technology, Danvers, USA), diluted in PBS/1% goat serum/1% donkey serum/1% BSA. Secondary antibodies: donkey anti-mouse Alexa Fluor 594, 1:1000 (A21203, Invitrogen via Life Technologies, Darmstadt, Germany), goat anti-rabbit Oregon Green 488, 1:1000 (O-6381, Invitrogen), diluted in PBS/1% goat serum/1% donkey serum/1% BSA.

Solutions: 4 % paraformaldehyde: For 250 ml stock solution 10 g paraformaldehyde are dissolved in 250 ml 1x PBS and the pH is adjusted to 7.4. The solution is heated to 65°C and NaOH is added until the paraformaldehyde is completely dissolved. Glycine solution: 100 mM in 1xPBS; pH is adjusted to 7.4 Blocking solution: 10 % goat serum, 10% donkey serum, 1% BSA (Capricorn Scientific, Ebsdorfergrund, Germany) in 1x PBS. DAPI stock solution: 10 mg/ml in water, DAPI working solution: 1 µg/ml (dilute 1 µl DAPI stock solution in 10 ml PBS).

**Calcium imaging in HL-1 cells**

Calcium homeostasis in HL-1 cells was measured by ratiometric fluorescence microscopy using Fura-2 AM. Changes in intracellular (cytosolic) calcium were measured at single-cell level using the VisiChrome High Speed Polychromator System (with High Speed Random Access Monochromator; Visitron Systems, Puchheim, Germany) and the Observer A.1 Axio inverse fluorescence microscope (Zeiss, Oberkochen, Germany) connected to a CoolSNAP EZ CCD camera (Visitron Systems). HL-1 cells were plated on FCS-coated custom-made glass coverslips for 24 h in Claycomb medium with supplements. To enhance coating and adherence the glass coverslips were pre-treated with 1 M HCl. The next day, the cells transfected with miRCURY LNA miR-221/222 mimics or mimic negative control in DMEM as described above. After 24 h of transfection, cells were allowed to grow in Claycomb medium including supplements for further 24 h. After that, they were serum-starved for 24 h prior to calcium measurement. HL-1 cells were loaded with 4 µM Fura-2 AM (stock solution: 1 mM in DMSO) at 37°C for 30 min. During the experiment cells were superfused with pre-warmed control and test solutions (temperature when reaching the cells: 37°C). Ringer buffer (control solution) contained in mM: NaCl 122.5, KCl 5.4, MgCl_2_ x 6 H_2_O 0.8, CaCl_2_ x 2 H_2_O 1.2, NaH_2_PO_4_ x H_2_O 1.0, glucose 5.5, HEPES 10; pH was adjusted to 7.4 at 37°C. Before beginning the measurement, cells were allowed to get accustomed to the superfusion with Ringer solution for 200 s. The measurement started with control solution for 200-300s to get a stable baseline signal, followed by superfusion with 100 nM AngII for 150 s to observe a transient [Ca^2+^]_i_ increase. After this, control Ringer solution was applied for 150 s to wash out AngII. To depolarize the cell membrane and activate LTCCs, the next step included Ringer solution with 25 mM KCl (instead of 5 mM in control solution) for 300 s. In a last step, cells were superfused with 1 µM ionomycin which serves as a positive control to discriminate between non-responding and dead cells. Living cells that do not respond to AngII and/or KCl still react to ionomycin with an increase in cytosolic Ca^2+^ concentrations. Dead cells on the other hand no longer have an intact plasma membrane, leading to an early accumulation of calcium in the cytosol and therefore no pronounced response upon ionomycin application. To test whether AngII and 25 mM KCl elicit the expected specific effects, inhibitors of AT1R (losartan, 10 µM) and LTCC (verapamil, 20 µM) were applied in additional experiments. Images of Fura-2 fluorescence intensity at excitation wavelengths of 340 nm and 380 nm were obtained with VisiVIEW Imaging Software (Visitron Systems; exposure: 40 ms, bin: 2, sampling interval: 2 s, emission: 510 nm). Analysis was performed on single cell level by defining one region of interest per cell. 340/380 ratios were exported to Excel. Background fluorescence was subtracted. The reaction of a cell to a substance was defined as a response if the maximum value was higher than the mean plus three times the standard deviation of the control solution before substance application (e.g. maximum value (AngII) > mean (Ringer) + 3x standard deviation (Ringer)). First, values from dead cells were eliminated due to lack of response to ionomycin. Then, responding and non-responding cells were defined. Response parameters, e.g. baseline shift, peak height, AUC (AngII: 150 s, KCl: first 150 s), were only obtained from responding cells unless indicated otherwise. The AUC was calculated using the trapezoid method: (x2-x1)*[((y1+y2)/2)-baseline]. The area was calculated for each time interval (2 s each) and then added. The AUC was calculated for the first 150 s after start of 25 mM KCl superfusion, not from the beginning of an individual response up to the corresponding maximum response.

**Calcium measurement in neoCM**

Calcium transients from spontaneous or electrically evoked activity in neoCM were obtained with the Myocyte Calcium and Contractility System (IonOptix, Westwood, Massachusetts, USA). This system provides a Galvanometer-driven mirror to switch between two wavelengths (340 nm, 380 nm) at a frequency of up to 250 Hz, allowing for very high sampling rates up to 250 Hz. NeoCM were measured 6-9 days after isolation. NeoCM monolayers were transiently transfected with miR mimics for 24 h as described above. NeoCM were kept on 1% FCS for further 48 h until the measurement. Cells were loaded with 4 µM Fura-2 AM (stock solution: 1 mM in DMSO) at 37°C for 30 min. After washing the monolayers once with medium, they were incubated with medium for 15 min at 37°C to allow complete de-esterification of internalized Fura-2. Cells were washed in Ringer solution (pH 7.4 at room temperature; composition see above) and measured at room temperature or stored at room temperature until the measurement. Each sample was measured three times: after recording control signals for 2 min the buffer was changed to 10 µM isoprenaline (ISO) (2 min break) and response to ISO was measured for 3 min and after a 1 min break again for 3 min. If possible, spontaneous calcium transients were recorded. Otherwise, cells were paced at 1 Hz to evoke calcium transients (MyoPacer, 5-10 V per pulse, pulse duration maximum 4 ms). IonWizard 6.6 (IonOptix) was used for data acquisition at sampling frequencies of 100 Hz (average 4: 4 collected data points are averaged into one raw data point) or 250 Hz (average 1). Monotonic transient analysis was performed using IonWizard 6.6. Prior to analysis, raw data were filtered with the lowpass Butterworth filter (cutoff frequency: 5 Hz, number of poles: 2). Transient marks were converted from event marks of the MyoPacer (transient values: offset: -0.05 s, duration: 0.9 s). T_0_ is defined as the time of the event mark for paced cells. For spontaneous transients transient marks were added manually and the time of departure was chosen as T_0_. Every transient was manually checked to assure appropriate detection and recognition of peaks and fitting of transient parameter functions. An overview of the applied transient parameters is given in Fig. 3B. Average calcium transients were obtained with IonWizard to visualize differential effects of ISO on miR-transfected monolayers.

**Contraction analysis in neoCM**

NeoCM were isolated as described above and cells from about one heart were plated onto one 35 mm petri dish. Monolayers were transfected with mimic control or miR-222 as described above and measured after about 7-8 days in culture. Before the recording cells were washed with Ringer buffer once. Cells were then placed into a pre-heated Ibidi chamber (Ibidi heating system 1; plate temperature: 38°C, lid: 42°C; Ibidi, Gräfelfing, Germany) and allowed to acclimatize for 1 min. Contraction recordings were performed as follows: acclimatization of the cells in Ringer for 60 s, movie 1 (control) 30 s, buffer change to 10 µM ISO and acclimatization 150 s, movie 2 (ISO 1) 30 s, break 90 s, movie 3 (ISO 2) 30 s. The following recording parameters were applied: 10x objective, Observer A.1 Axio inverse fluorescence microscope (Zeiss) connected to a CoolSNAP EZ CCD camera (Visitron Systems); sampling rate: 10 fps; VisiView Software. All three movies from the same petri dish recorded the same section of cells. Movies (.avi) were generated without compression and with a frame rate of 10 fps.

Movies were analyzed using Myocyter [25] with the following parameters: “detection”: 10, “% of max recognized as beat”: 20. Manual ROIs were carefully placed to analyze the same regions in all three movies from one petri dish. Number of ROIs varied between 8-37 depending on confluency and synchronicity of the monolayer and cluster size within the monolayer. The number of spontaneous contractions for each ROI within each 30 s movie was extracted using the parameter “beats counted”, always excluding the first and last event.
