# Supplementary figures and images for: Influence of miR-221/222 on cardiomyocyte calcium handling and function
Source: Cell Biosci. 2021 Aug 17;11:160. doi: 10.1186/s13578-021-00676-4 (PMC8369661; doi:10.1186/s13578-021-00676-4)

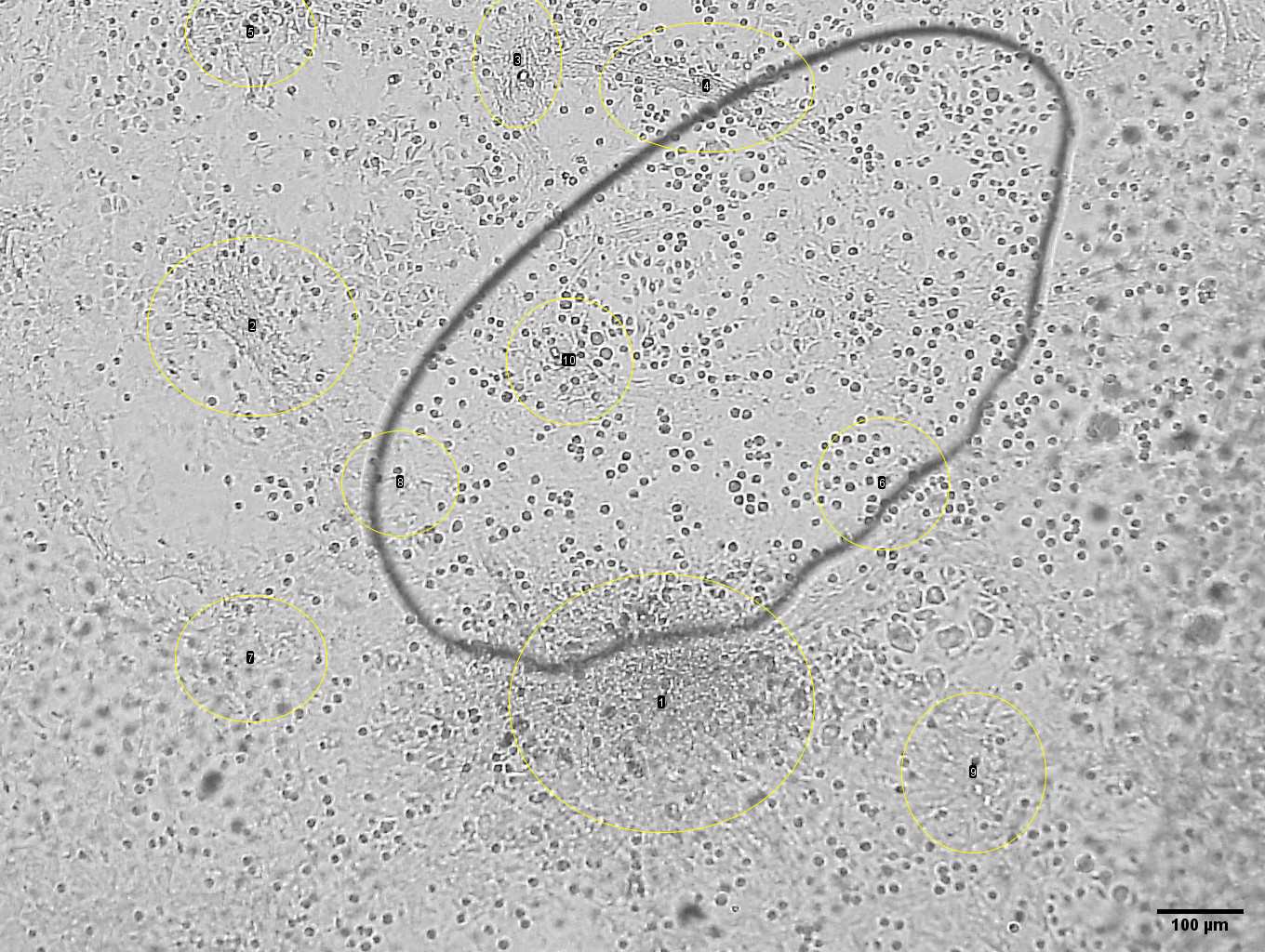

Supplement: Supplementary file 2 — Additional file 2: Figure S2. ROI overview: Untransfected neoCM spontaneous contraction control (for quantification see Additional file 1: Figure S1). [file 13578_2021_676_MOESM2_ESM.jpg]

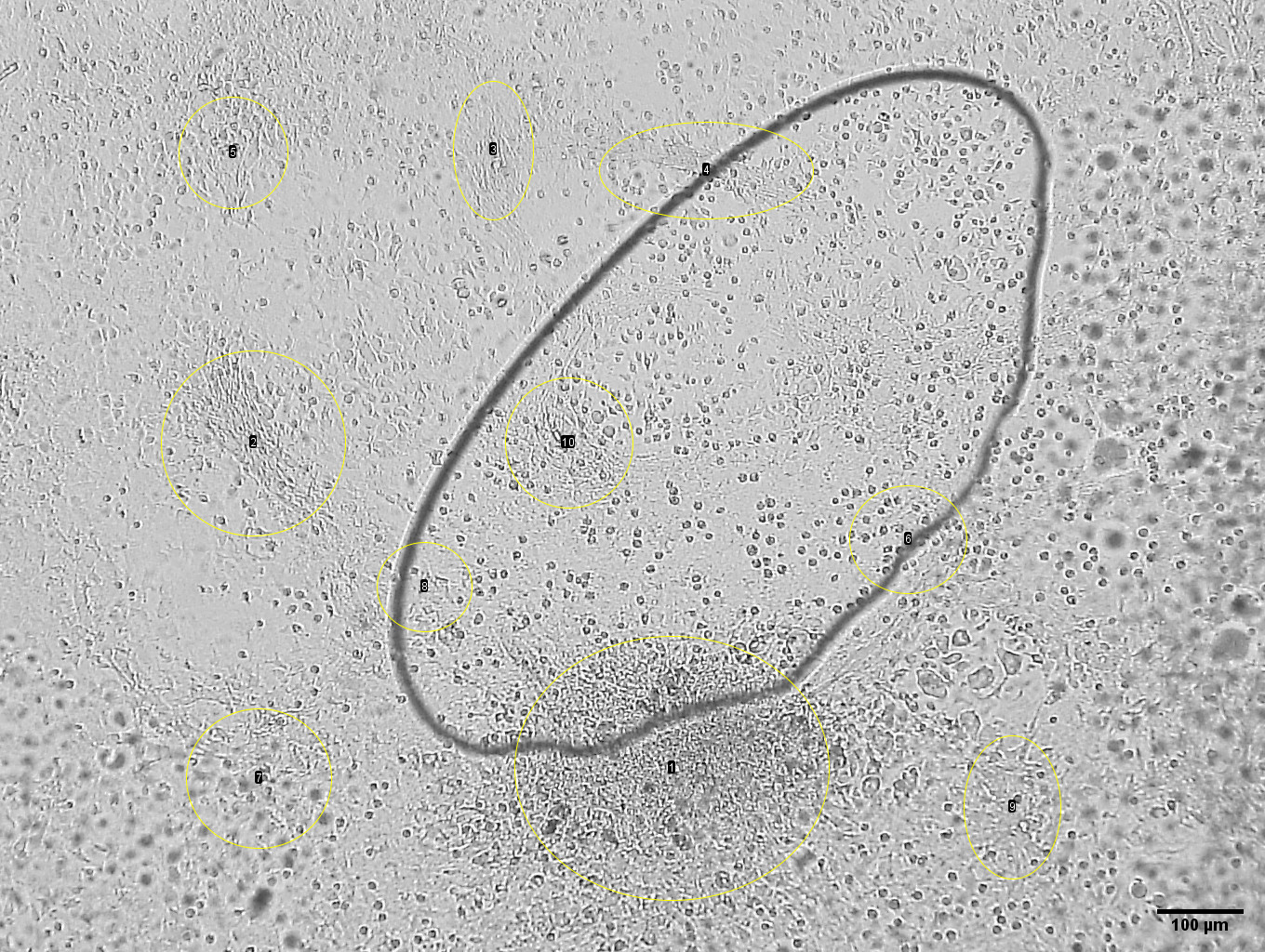

Supplement: Supplementary file 3 — Additional file 3: Figure S3. ROI overview: Untransfected neoCM spontaneous contraction verapamil (for quantification see Additional file 1: Figure S1). [file 13578_2021_676_MOESM3_ESM.jpg]

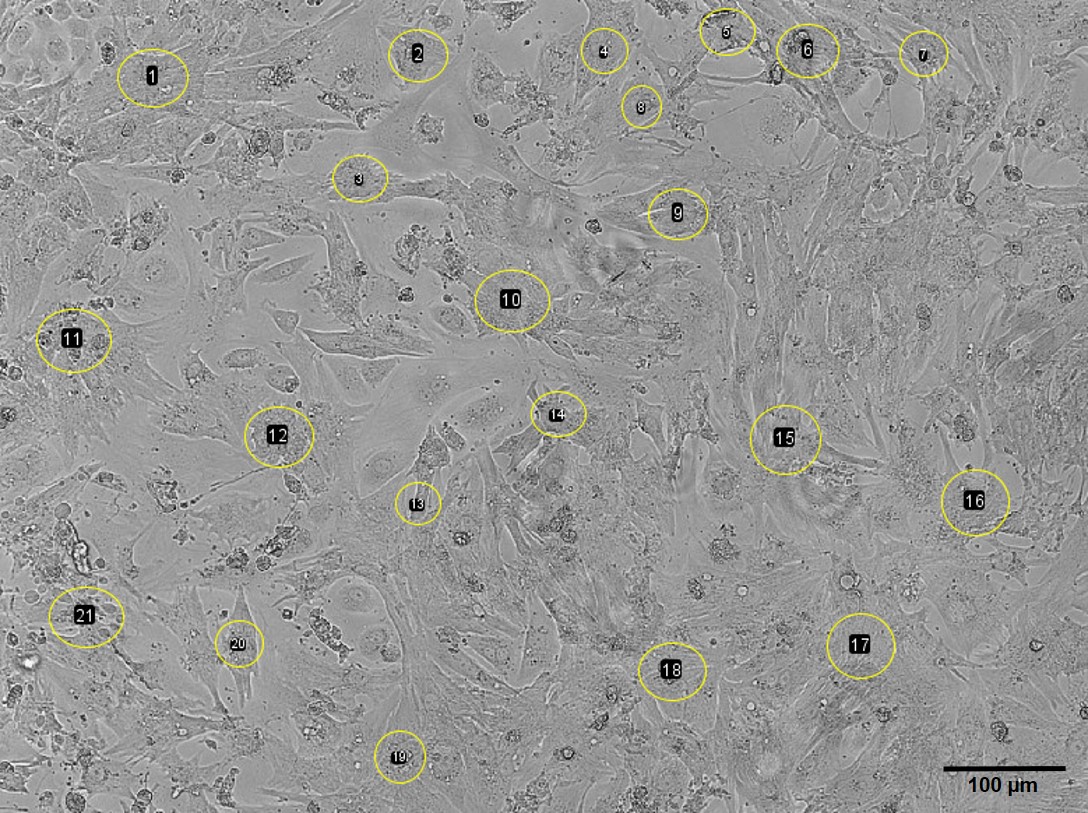

Supplement: Supplementary file 4 — Additional file 4: Figure S4. ROI overview: mc-transfected neoCM spontaneous contraction control (for corresponding tracings of ROI 3 see Fig. 5A). [file 13578_2021_676_MOESM4_ESM.jpg]

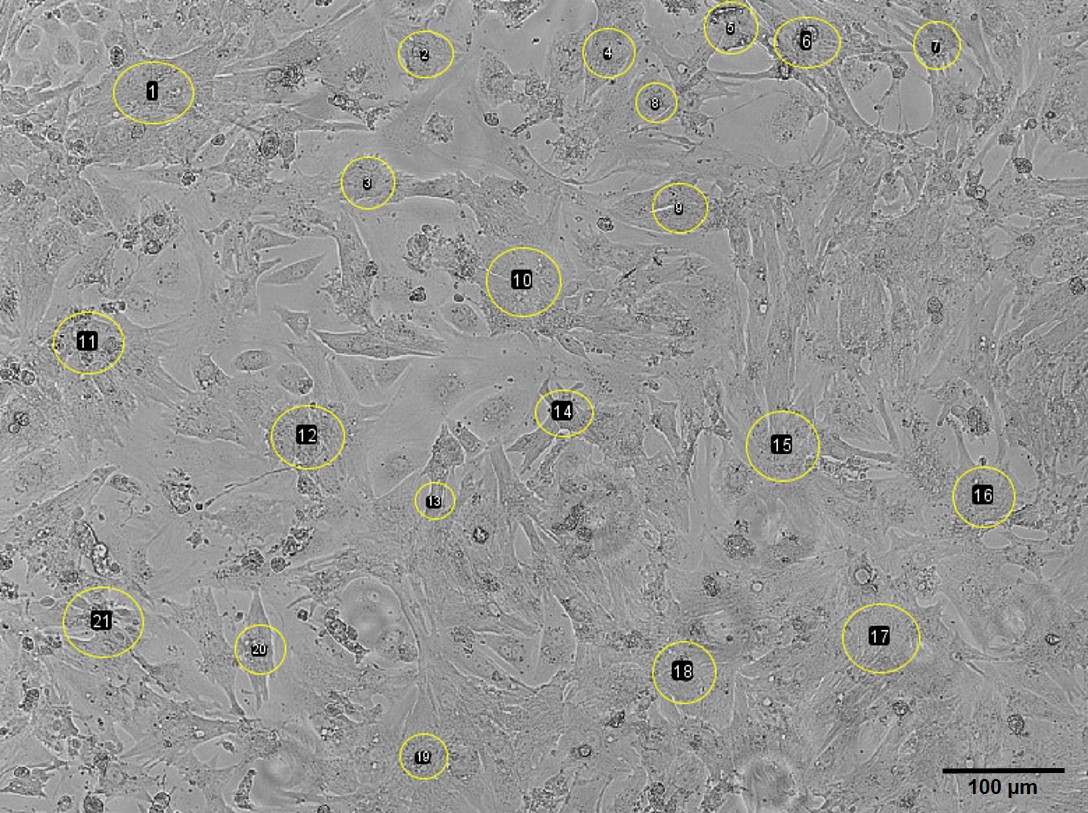

Supplement: Supplementary file 5 — Additional file 5: Figure S5. ROI overview: mc-transfected neoCM spontaneous contraction ISO 1 (for corresponding tracings of ROI 3 see Fig. 5A). [file 13578_2021_676_MOESM5_ESM.jpg]

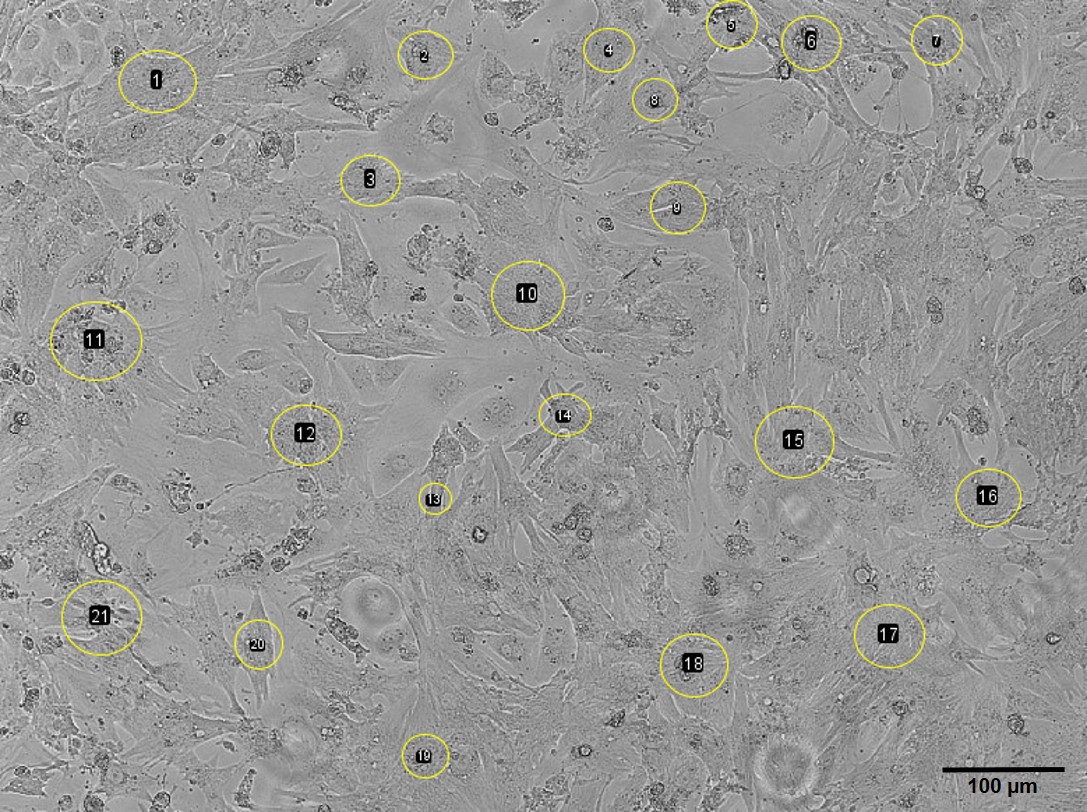

Supplement: Supplementary file 6 — Additional file 6: Figure S6. ROI overview: mc-transfected neoCM spontaneous contraction ISO 2 (for corresponding tracings of ROI 3 see Fig. 5A). [file 13578_2021_676_MOESM6_ESM.jpg]

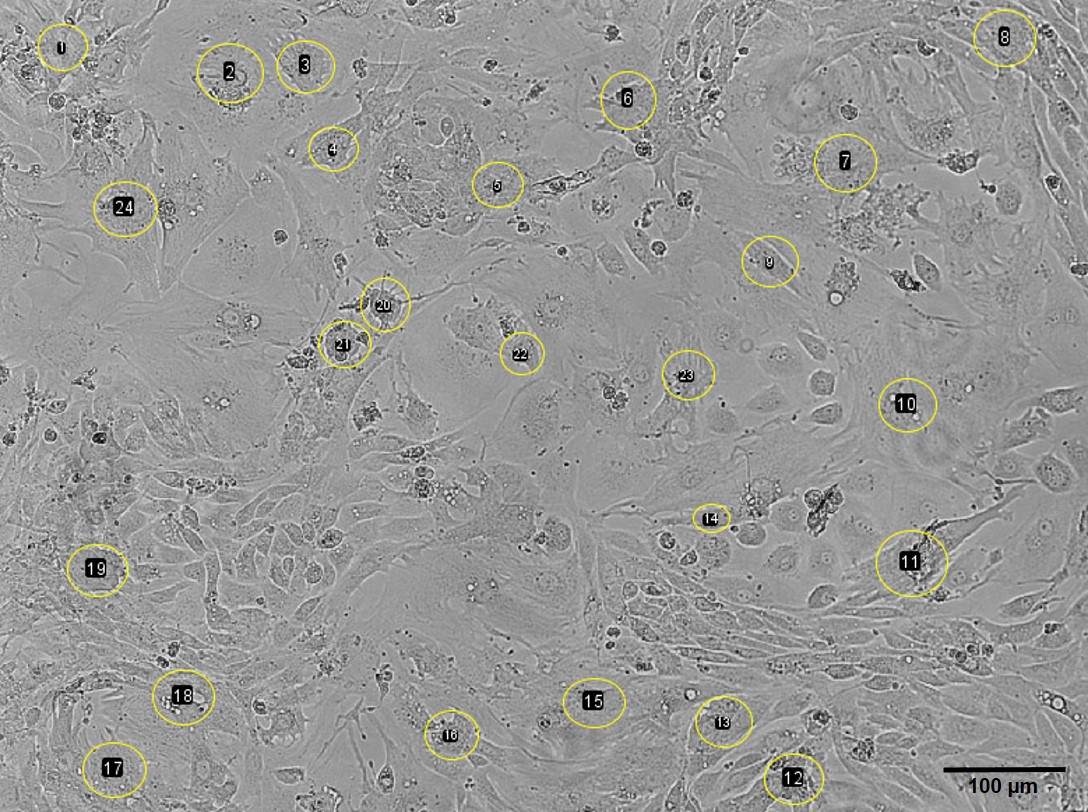

Supplement: Supplementary file 7 — Additional file 7: Figure S7. ROI overview: miR-222-transfected neoCM spontaneous contraction control (for corresponding tracings of ROI 1 see Fig. 5A). [file 13578_2021_676_MOESM7_ESM.jpg]

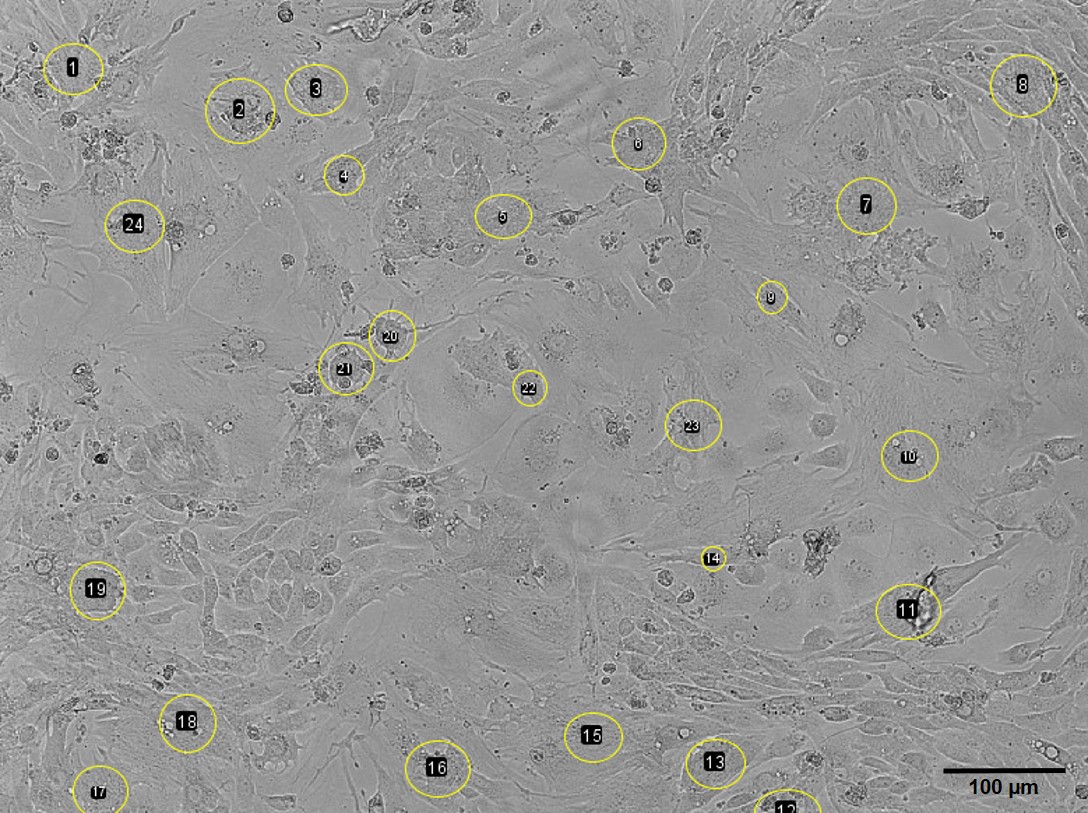

Supplement: Supplementary file 8 — Additional file 8: Figure S8. ROI overview: miR-222-transfected neoCM spontaneous contraction ISO 1 (for corresponding tracings of ROI 1 see Fig. 5A). [file 13578_2021_676_MOESM8_ESM.jpg]

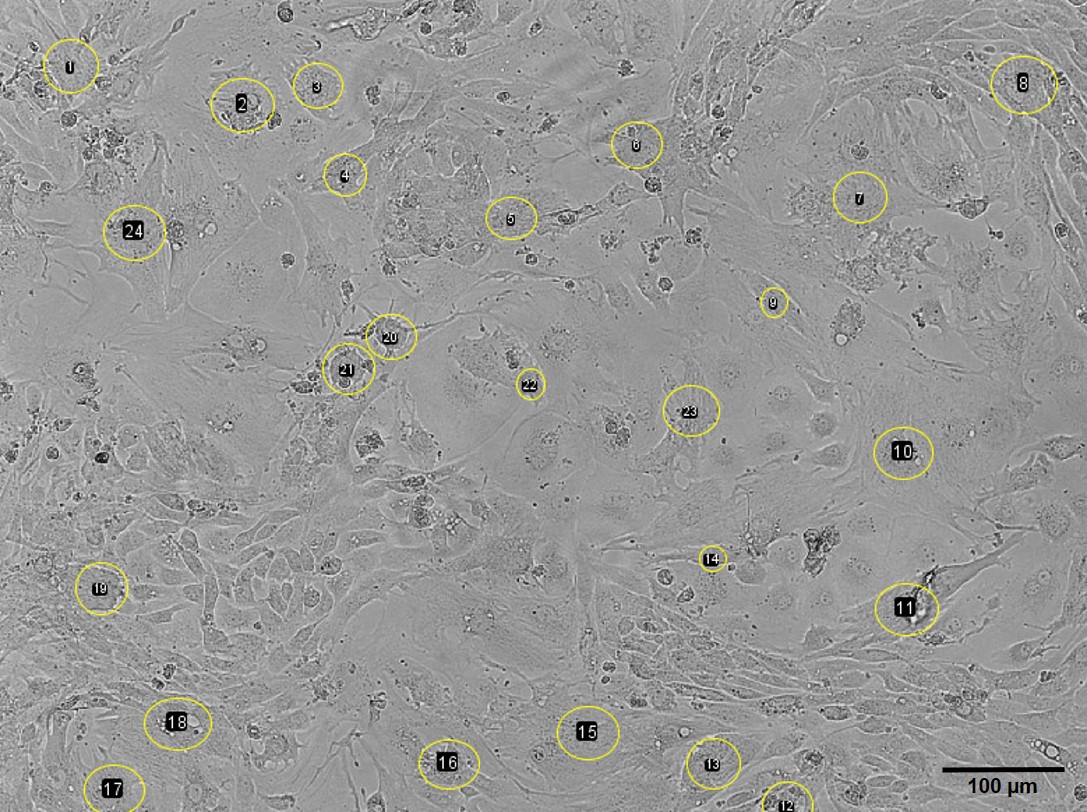

Supplement: Supplementary file 9 — Additional file 9: Figure S9. ROI overview: miR-222-transfected neoCM spontaneous contraction ISO 2 (for corresponding tracings of ROI 1 see Fig. 5A). [file 13578_2021_676_MOESM9_ESM.jpg]
